# Multiplex Genome-Editing Technologies for Revolutionizing Plant Biology and Crop Improvement

**DOI:** 10.3389/fpls.2021.721203

**Published:** 2021-10-06

**Authors:** Mohamed Abdelrahman, Zheng Wei, Jai S. Rohila, Kaijun Zhao

**Affiliations:** ^1^National Key Facility for Crop Gene Resources and Genetic Improvement, Institute of Crop Science, Chinese Academy of Agricultural Sciences, Beijing, China; ^2^Rice Research and Training Center, Field Crops Research Institute, Agricultural Research Center, Kafr El-Shaikh, Egypt; ^3^Dale Bumpers National Rice Research Center, United States Department of Agriculture - Agricultural Research Services, Stuttgart, AR, United States

**Keywords:** multiplex genome editing, CRISPR/Cas9, CRISPR/Cas12, plant science, crop improvement

## Abstract

Multiplex genome-editing (MGE) technologies are recently developed versatile bioengineering tools for modifying two or more specific DNA loci in a genome with high precision. These genome-editing tools have greatly increased the feasibility of introducing desired changes at multiple nucleotide levels into a target genome. In particular, clustered regularly interspaced short palindromic repeats (CRISPR)/CRISPR-associated protein (Cas) [CRISPR/Cas] system-based MGE tools allow the simultaneous generation of direct mutations precisely at multiple loci in a gene or multiple genes. MGE is enhancing the field of plant molecular biology and providing capabilities for revolutionizing modern crop-breeding methods as it was virtually impossible to edit genomes so precisely at the single base-pair level with prior genome-editing tools, such as zinc-finger nucleases (ZFNs) and transcription activator-like effector nucleases (TALENs). Recently, researchers have not only started using MGE tools to advance genome-editing applications in certain plant science fields but also have attempted to decipher and answer basic questions related to plant biology. In this review, we discuss the current progress that has been made toward the development and utilization of MGE tools with an emphasis on the improvements in plant biology after the discovery of CRISPR/Cas9. Furthermore, the most recent advancements involving CRISPR/Cas applications for editing multiple loci or genes are described. Finally, insights into the strengths and importance of MGE technology in advancing crop-improvement programs are presented.

## Introduction

Plant scientists have long been searching for biological tools to generate site-specific mutations in the plant genome for research. Until the emergence of genome-editing tools, it was almost impossible to recognize and alter specific genomic sequences at a chosen locus. Recently, nucleases have been engineered that recognize and cleave DNA at target genomic sites, introducing double strand breaks (DSBs) that are subsequently repaired by either error-prone non-homologous end joining (NHEJ) or homology-directed repair (HDR) pathways. During the repair process, precise DNA fragment deletion, insertion, or replacement can occur, resulting in gene editing, mutation, or replacement (Hsu et al., 2013; Miglani, 2017; Mishra et al., 2018).

Using nucleases to simultaneously and precisely introduce two or more DSBs at specific loci within a single genome in a single round of mutagenesis will hereafter be called multiplex genome editing (MGE) (Cong et al., 2013). MGE generates rapid desirable changes in specific but different genes or quantitative trait loci (QTLs), resulting in the development of new plant genotypes carrying multiple mutations in one generation. The capability to create such genomic changes will allow researchers to understand and study the interactions between members of a gene family that have redundant functions, and it may provide an opportunity to analyze epistatic relationships among closely related genetic pathways (Xing et al., 2014). Furthermore, these multiple genomic modifications or edits could provide the most practical platform for metabolic pathway engineering in plants. MGE can be used to facilitate many desirable crop improvements, such as the production of seedless plant species and better disease resistance (Naim et al., 2018; Tripathi et al., 2019), as well as to skip extensive segregation in vegetatively propagating crop plants, such as sugarcane (Kannan et al., 2018).

Unlike traditional random mutagenesis, MGE tools are designed to target specific genomic regions and to generate specific mutations or variations with much more precision (Belhaj et al., 2015). Accomplishing multiple gene mutations in an elite cultivar formerly required many years and several cycles of crossing and selection. Moreover, the traditional approach for conventional breeding has sometimes been more complicated because of linkage drag, which impedes the transmission of good traits aside from deleterious genes. For instance, developing high-yielding genotypes with premium grain quality traits has always been considered a challenge for plant breeders utilizing conventional breeding approaches (Abdelrahman and Zhao, 2020). However, many researchers have reported using MGE tools to improve traits with such complications (Wang et al., 2014; Li W. et al., 2016; Kannan et al., 2018). MGE tools have become more user-friendly, particularly after the development and adaptation of the CRISPR-Cas system (Jinek et al., 2012; Cho et al., 2013; Cong et al., 2013; Mali et al., 2013). Among genome-editing technologies, including zinc-finger nucleases (ZFNs) (Bibikova et al., 2002; Lloyd et al., 2005; Ainley et al., 2013; Qi et al., 2014) and transcription activator-like effector nucleases (TALENs) (Christian et al., 2010; Li et al., 2011), the CRISPR/Cas system uniquely depends on base complementarity between the guide RNA (gRNA) and the target DNA for recognition, which greatly simplifies the genome-editing process (Jinek et al., 2012). Due to its simplicity for designing multiple target genetic loci, researchers have preferred the CRISPR/Cas system for genome editing in recent years. TALENs have also been successfully utilized for MGE in polyploid plants, including wheat, tobacco, and sugarcane (Wang et al., 2014; Li W. et al., 2016; Kannan et al., 2018). However, while using the multiplex TALENs approach, each unit must be designed separately, which increases the difficulty in delivering different units simultaneously to a plant cell. The CRISPR/Cas system has many advantages over TALENs and ZFNs: it is more efficient in editing target loci, and multiple single-guide RNAs (sgRNAs) can be designed to be expressed at once for simultaneous editing of multiple loci (Cong et al., 2013). Adding more gRNAs to the transformation construct is far simpler than engineering new proteins to improve the trait of choice in plants. By using just one Cas protein with multiple gRNAs, multiple gene mutations have been achieved in various plants, including rice, wheat, maize, tomato, and *Arabidopsis* (Li et al., 2013; Upadhyay et al., 2013; Ma et al., 2015; Yan et al., 2016; Rothan et al., 2019).

Further investigations and developments are ongoing to improve MGE applications for the genetic improvement of crops. To date, MGE using CRISPR/Cas has been efficiently conducted to enhance crop yield, quality, and stress resistance (Xu et al., 2016; Kaur et al., 2018; Li Y. et al., 2019; Zhou et al., 2019). The results of different experiments have shown that different applications and procedures are progressively appearing. Immense technical progress has recently occurred in this research field, utilizing more complex genetic, epigenetic, and transcriptional manipulations for research needs. Recently, Kannan et al. (2018) reported targeted mutagenesis of more than 100 alleles in sugarcane to improve saccharification efficiency without affecting biomass yield. In this review, we discuss different emerging applications that have been developed and adapted for MGE and their potency and associated experiments for crop-improvement programs. We further summarize and provide recent information about the progress on MGE in plants, emphasizing its role in developing plant molecular biology and plant breeding.

## Tools for MGE

### TALENs for Targeting Gene Families

When MGE tools were first developed, before there was a CRISPR/Cas system, Wang et al. (2014) reported using a single TALEN pair to edit three homoeoalleles encoding mildew resistance locus proteins (MLOs) in hexaploid bread wheat (*Triticum aestivum* L., 2n = 42, AABBDD). The goal was to develop broad-spectrum and durable *Blumeria graminis* f. sp. *tritici* (*Bgt*) resistant wheat lines. The single TALEN pair recognition sequences were strictly conserved at the *MLO-B1* and *MLO-D1* target sites and has one nucleotide mismatch at the *MLO-A1* target site; they had mutations at the three target sites. The results revealed that of the 27 T0 plants, four mutants carried two edited targets in either homozygous or heterozygous forms, and only one plant harbored triple mutations. Plants with triple mutations at the three different targets showed resistance, with no apparent fungal growth on the leaves, although abundant fungal growth was found on the leaves of wild-type plants, and the plants with combinations of other mutations.

Multiplex editing using TALENs was also employed to produce highly potent biopharmaceutical glycoprotein products. In an attempt to increase their activity, stability, and immunogenicity in plants, Li J. et al. (2016) reported that two α *(1,3)-fucosyltransferase* (*FucT1* and *FucT2*) and two β *(1,2)-xylosyltransferase* (*XylT1* and *XylT2*) genes within *Nicotiana benthamiana* were successfully edited, resulting in plants with improved capacity to produce glycoproteins devoid of plant-specific residues. Conserved sequences in the first exon of each gene family were analyzed, and one sequence-specific TALEN pair for each gene family was designed accordingly. The researchers tried to knockout the two-gene family members separately and together by cotransformation of the two TALEN pairs. Both *FucT* and *XylT* mutant plants had significant changes in their N-glycan profiles. Notably, this was the only trial of multiplex editing using two different modules of TALEN pairs to target two divergent gene families in one genotype.

To date, the highest record for simultaneous targeting genome editing in the same plant genotype has been reported in sugarcane crops. Kannan et al. successfully edited 107 of 109 gene copies of the sugarcane *caffeic acid O-methyltransferase* (*COMT*) gene family by introducing a single TALEN pair (Kannan et al., 2018). A conserved region of *COMT* was targeted with a single TALEN pair for multiallelic mutagenesis to modify lignin biosynthesis in sugarcane. This improved saccharification efficiency by up to 43.8% and significantly reduced lignin content in the mutant, without significant differences in biomass production compared with the wild type.

These studies made it clear that TALEN-based genome editing allows for efficient multiplex editing of gene families with conserved sequences among different gene copies, and this has been successfully demonstrated in polyploid genomes. However, Li et al. succeeded in editing two different gene families with two different cotransformed TALEN pairs; the editing efficiency of several TALEN pairs for each target was tested before conducting the experiment (Li J. et al., 2016). Furthermore, the procedure is more complicated if more gene families are targeted simultaneously because TALEN technologies require the engineering of DNA-binding domains for individual targeting applications (Lowder et al., 2015).

### The CRISPR/Cas9 System Offers Excellent MGE Ability

The CRISPR/Cas9 is much easier and more efficient than other genome-editing tools, particularly for the editing of multiple unrelated genes. Using multiple sgRNAs and expressing only one Cas protein significantly simplifies and enhances the editing efficiency of multiple genes. The ease of plasmid construction in CRISPR/Cas9 experiments further allows the function of multiple associated genes to be investigated that either has negative effects or that improve plant-specific characteristics.

Attempts have been made to improve the CRISPR/Cas system to further simplify and enhance the efficiency of editing multiple genes. The core focus has been to develop an easy and fast toolbox to insert multiple sgRNA expression cassettes into one binary vector. Several sequential rounds of regular cloning steps can be utilized to insert different expression cassettes containing sgRNAs for different targets into a single construct, but this is very laborious and there is a limited number of assembled sgRNAs (Li et al., 2013; Mao et al., 2013; Zhou et al., 2014; Yan et al., 2015). A more advanced cloning method, called Golden Gate cloning, depends on the use of type IIS restriction enzymes in the DNA assembly. Golden Gate cloning has been used to generate non-palindromic sticky ends in DNA fragments carrying sgRNAs. This technique facilitates the cloning of different constructs into binary vectors based on the different toolkits available for CRISPR/Cas9-mediated genome editing. The methods investigated to enhance the cloning of different gRNAs into a single binary vector can be divided based on the technique used to assemble the sgRNAs together in the single delivery construct. These strategies are either based on assembling multiple individual gRNA expression cassettes, each transcribed from RNA polymerase III promoters, such as U6 or U3 (Xing et al., 2014; Lowder et al., 2015; Ma et al., 2015; Wang C. et al., 2015; Yan et al., 2015; Vazquez-Vilar et al., 2016; Zhang et al., 2016; Zhao et al., 2016; Char et al., 2017; Ordon et al., 2017; Tian et al., 2017), or through a single transcript of polycistronic mRNAs that are cleaved into individual gRNAs posttranscriptionally by the CRISPR-associated RNA endoribonuclease Csy4 from *Pseudomonas aeruginosa* (Tsai et al., 2014); the tRNA processing enzymes, naturally present in the host cells (Xie et al., 2015); or self-processing ribozymes (Gao and Zhao, 2014; Tang et al., 2016).

#### RNA Polymerase III Promoters Drive the Expression of Multiple sgRNAs

**Figure 1:**
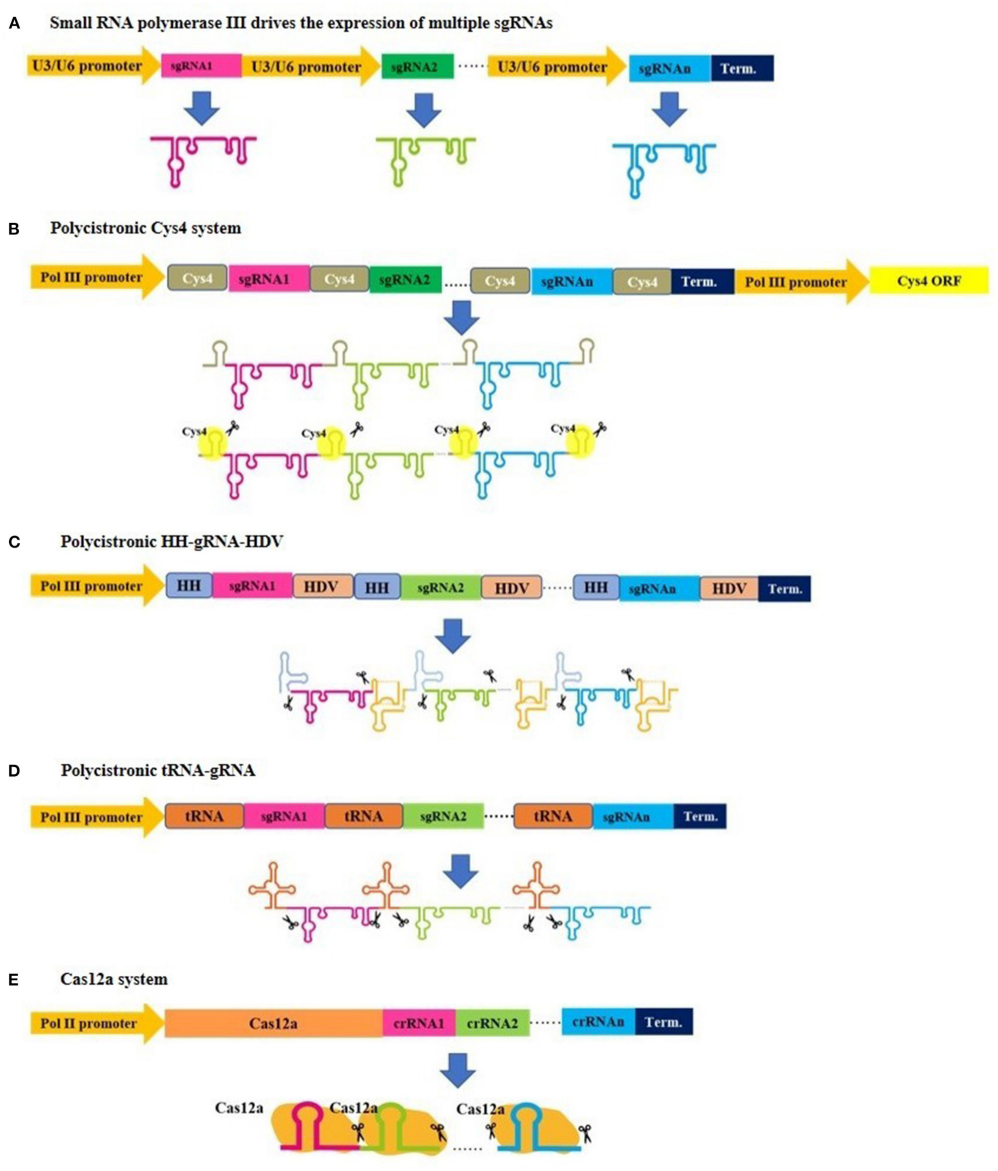
An illustration demonstrating various strategies for expressing multiplex gRNA cassettes in plants. **(A)** Small gRNAs are cloned after U3 or U6 promoters and derived by small RNA polymerase III to generate individual gRNAs. **(B–D)** Small gRNAs are cloned to be transcribed into a single transcript, and subsequent posttranscriptional processing is needed for gRNA separation, where Csy4, tRNAs, and hammerhead ribozyme regulate this separation. Similarly, a single transcript is generated in the **(E)** Cas12a system, but this system has a gRNA self-cleaving feature and does not require additional elements for posttranscriptional processing.

Two sets of CRISPR/Cas9 constructs have been developed by Xing et al. (2014) and Ma et al. (2015), where the gRNAs are driven by small RNA polymerase III (Figure 1A) and designed for cloning into a binary vector with prepared sgRNA expression cassettes in a single round of Golden Gate cloning using Type IIS restriction enzymes, such as *Bsa*I. This approach has allowed for simultaneous knockout of multiple genes in *Arabidopsis* and rice. Both sets of CRISPR/Cas9 constructs are the most commonly used tools of this type, and both require a specific promoter to drive each sgRNA. Similarly, Lowder et al. (2015) designed an approach for MGE that uses RNA polymerase III to drive sgRNA expression in a successive three-step procedure, which is as follows: an intermediate vector carrying the sgRNAs of interest is constructed first, followed by Type IIS restriction enzyme digestions, recovery of the sgRNA cassettes, and then Golden Gate cloning into a binary vector.

#### Single Transcript System for the Expression of Multiple sgRNAs

Although the abovementioned method in section RNA Polymerase III Promoters Drive the Expression of Multiple sgRNAs is frequently used for MGE, one limitation is the size of the final construct, which can sometimes be too large and repetitive due to repeating promoter sequences. These repeats can induce frequent recombination events (Vidigal and Ventura, 2015; Ding et al., 2020) and cause plasmid instability in *E. coli* and *Agrobacterium*. This might be why this method has been reported to result in the assembly of two to eight gRNA expression cassettes. Recently, Stuttmann et al. (2021) reported a new CRISPR system to allow multiplexing of up to 24 gRNAs in *Arabidopsis*; they observed that processing polycistronic single transcripts containing multiple gRNAs has a major advantage because they can be produced by a polymerase II promoter, which can produce longer transcripts than a polymerase III promoter (Arimbasseri et al., 2013).

In an early attempt to have a polycistronic CRISPR/Cas9 system for MGE, Tsai et al. developed a vector in which multiple gRNAs can be assembled, each flanked by cleavage sites for the Csy4 RNase of *Pseudomonas aeruginosa* (Haurwitz et al., 2010; Tsai et al., 2014). Based on this, each gRNA must be designed to have a 3′ end with a Csy4 recognition site to allow the coexpressed Csy4 to cleave the long transcript, thereby releasing the gRNAs (Figure 1B).

Mimicking the native tRNA-snoRNA43 dicistron (Kruszka et al., 2003) in cells, Xie et al. further designed a polycistronic tRNA-gRNA (PTG) system for simultaneous production of single gRNA transcripts (Figure 1C), in which the gRNAs are transcribed by RNA polymerase III and linked together by their tRNA sequences (Xie et al., 2015). The tRNAs are then recognized and processed by endogenous RNases (e.g., RNase P and RNase Z in plants) that excise the individual gRNAs from the transcript. These gRNAs direct Cas9 (driven by RNA polymerase II) to their targets for genome editing. This application has resulted in highly efficient genome editing, although Xie and his group observed a reduction in the efficiency of PTGs with an increasing number of gRNAs, possibly due to multiple sgRNAs competing for binding to Cas9.

In an alternative approach, Tang et al. designed a CRISPR/Cas system to allow the expression of both Cas9 and sgRNAs from a single Pol II promoter using a self-processing hammerhead ribozyme (Haseloff and Gerlach, 1988; Tang et al., 2016). The Cas9 and sgRNAs were linked by ribozyme (RZ) cleavage sites, allowing transcripts to be cleaved by the cis-acting ribozyme (Figure 1D). The approach was tested using six target sites in three rice genes, and the mutation frequencies in T0 plants ranged from 53.8 to 100% with a majority of biallelic mutations.

In the same context, Cermák et al. designed several multiplexed gene-editing tools based on the posttranscriptional processing of a single polycistronic mRNA using the CRISPR-associated Csy4 RNA endoribonuclease from *Pseudomonas aeruginosa* (Tsai et al., 2014; Cermák et al., 2017), the tRNA processing enzymes naturally present in the host cells (Xie et al., 2015), and ribozymes (Gao and Zhao, 2014). Furthermore, Cermák and colleagues compared the editing efficiency of these systems for targeting two sites in the *AUXIN RESPONSE FACTOR 8A* (*ARF8A*) gene of tomato, and found that Csy4 had the best efficiency, followed by the tRNA system. The trial results were confirmed with eight gRNAs and in four different tomato genes. The efficiency of the Csy4 system was further demonstrated using up to 12 gRNAs for targeted gene deletions.

### The CRISPR/Cas12a (Cpf1) System: An Emerging Tool for MGE

For the aforementioned CRISPR/Cas9 systems, the size of the final construct is one of the most critical limitations in multiplexing experiments. Moreover, the requirement of GC-rich protospacer-adjacent motif (PAM) sequences is a drawback of using Cas9, especially when the number of genes to be edited is large. Furthermore, even in cases where single polycistronic multiple gRNA methods are employed, additional sequences must be added for further processing after transcription. These limitations may be overcome by a newer member of the Cas protein family, Cas12a, formerly known as Cpf1 (Zetsche et al., 2015). Cas12a has a relatively small size, its required PAM sequence allows T-rich regions to be targeted, and it requires a short crRNA (42 nt) which facilitates construction and multiplexing (Figure 1E). Moreover, the Cas12a multiplexing system has major advantages over other DNA editing systems. The Cas12a system does not require either the addition of several pol III promoters (only one Pol III promoter is enough to drive several small crRNAs) or the addition of specific sequences for multiple crRNA constructs because it can sufficiently process and mature crRNAs using its ribonuclease activity (Zetsche et al., 2015, 2017; Fonfara et al., 2016). These features make Cas12a an ideal system for MGE. Moreover, Cas12a cleaves the DSB at cohesive ends distal from the PAM site, which allows continuous cleavage of DNA (Zetsche et al., 2015). Swarts and Jinek compared the structure function relationships of Cas9 and Cas12a and their implications in genome editing in detail (Swarts and Jinek, 2018). Importantly, Cas12a was found to be very specific in plant MGE using whole-genome sequencing (Tang et al., 2018). Similar findings were reported while using CIRCLE-seq (Tsai et al., 2017; Lee et al., 2019).

### Inactive Cas Proteins for MGE

Mutating specific amino acids in Cas9 and Cas12a abolishes the DNA cleavage activity of these proteins, converting them into inactive (or dead) Cas (dCas). Fusion of dCas with cytosine deaminase (CBE) or adenosine deaminase (ABE) and sgRNAs induces point mutations at the target site. Editing a single nucleotide may be sufficient to alter gene structure to obtain a desirable function (Komor et al., 2016; Eid et al., 2018; Mishra et al., 2020). Shimatani et al. used such technology to induce multiple herbicide-resistance point mutations in rice (Shimatani et al., 2017). Recently, Xu et al. increased the efficiency of this technique by 2- to 5-fold by developing a tool kit that uses high-fidelity dCas9 variants fused to a tandemly arrayed tRNA-modified sgRNA architecture in rice (Xu et al., 2019). Furthermore, oriented transcriptional regulation was achieved by the fusion of dCas enzymes to effector domains, including CRISPR-mediated inhibitor (CRISPRi) and activator (CRISPRa) (Gilbert et al., 2013; Qi et al., 2013). Lowder et al. (2017) described a toolbox for constructing and cloning multiplex CRISPR/Cas9 transcriptional activation and the repression of *Arabidopsis* genes.

## Revolutionizing Roles of MGE in Plant Biology

CRISPR/Cas is bringing about a marked transformation in plant biology. MGE using CRISPR/Cas endows scientists with the ability to decipher very difficult biological problems by targeting different genes simultaneously. In this section, of the review, we aim to explore the impacts of the multiplex CRISPR/Cas system. Table 1 summarizes the efforts taken using MGE for revolutionizing plant biology and crop improvement.

**Table 1:** Summary presentation of the efforts done using multiplex genome editing for revolutionizing plant biology and crop improvement programs.

**MGE application**	**Target genes/QTLs**	**Crop**	**Objective**	**References**
**Plant biology**				
**Knock-out of multiple genes**				
	Two targets at ssDNA tomato yellow leaf curl virus (TYLCV) viruses	Tomato	Virus biological control in plants	Ali et al. (2015)
	*OsGW2, OsGW5*, and *OsTGW6*	Rice	Larger grain size	Xu et al. (2016)
	*RAS-PDS1* and *RAS-PDS2*	Banana	Demonstration of gene knockout in banana	Kaur et al. (2018)
	*OsSWEET11* and *OsSWEET14*	Rice	Broad-spectrum disease resistance in rice	Xu et al. (2019)
	*TMS5, Pi21*, and *Xa13*	Rice	Hybrid rice production and disease resistance	Li S. et al. (2019)
**Engineering efficient plant metabolic pathways**				
	*OsGSTU, OsMRP15, and OsAnP*	Rice	Anthocyanin synthesis pathway	Ma et al. (2015)
	Three different sites in the *OsWaxy*	Rice	Decreasing amylose content	Ma et al. (2015)
	Two sites in *slyPDS*	Tomato	Generating photobleached phenotype	Li R. et al. (2018)
	*GABA-TP1, GABA-TP2, GABA-TP3, CAT9*, and *SSADH*	Tomato	Metabolic engineering and manipulation of the gamma-aminobutyric acid (GABA)	Li R. et al. (2018)
	*SGR1, LCY-E, Blc, LCY-B1, and LCY-B2*	Tomato	Increase the accumulation of lycopene	Li X. et al. (2018)
	*GmF3H1, GmF3H2*, and *GmFNSII-1*	Soybean	Increasing the isoflavone content	Zhang et al. (2020)
**Transcriptional regulation**				
	Three different sites in the promoter of *production of Anthocyanin Pigment1* gene (*AtPAP1*, encoding a transcription factor) and *miR319* (encoding a microRNA)	*Arabidopsis*	Transcription gene activation	Lowder et al. (2015)
	Promoter regions of the *WRKY30, RLP23*, and *CDG1* genes	*Arabidopsis*	Simultaneous activation of their transcription	Li et al. (2017)
	Two sites at locule number (lc) QTL + eight gRNAs targeting the promoters of SlCLV3, S and SP	Tomato	Generating desirable/beneficial regulatory variants	Rodríguez-Leal et al. (2017)
	*OsWOX11* and *OsYUC1*	Rice	Transcription gene activation	Gong et al. (2020)
**Chromosomal segment restructuring**				
	Two targets flanking a 245 kb segment	Rice	Chromosomal segment deletions	Zhou et al. (2014)
	Two targets flanking 1.8 kb segment	Tobacco	Chromosomal segment deletions and inversion	Gao et al. (2015)
	Two targets flanking a 100 bp segment	*Arabidopsis*	Chromosomal segment deletions	Ordon et al. (2017)
	6 targets at a 58 kb region	Medicago	Chromosomal segment deletions	Cermák et al. (2017)
	Different genomic regions ranging from 1.7 to 13 kb	Arabidopsis	Chromosomal segment deletions	Wu et al. (2018)
	Two targets flanking an 18 (kb) segment	Arabidopsis	Chromosomal inversion	Schmidt et al. (2019)
**Multiplex base alterations**				
	Three sgRNAs targeting *ALS* and *FTIP1e genes*	Rice	Herbicide resistance	Shimatani et al. (2017)
	*OsACC*-T1, *OsALS-*T1, *OsCDC48*-T3, *OsDEP1*-T1, *OsDEP1*-T2, and *OsNRT1.1B-*T1.	Rice	Herbicide resistance	Li C. et al. (2018)
	*AtALS, AtPDS, AtFT*, and *AtLFY* genes and *BnALS* and *BnPDS*	Arabidopsis and Rapeseed	System verification	Kang et al. (2018)
	Two targets *StGBSSI* gene	Potato	impaired amylose biosynthesis in potato	Veillet et al. (2019)
	*ZmALS1* and *ZmALS2*	Maize	Herbicide resistance	Li Y. et al. (2019)
	Four targets at *OsWAXY, OsCDC48, and OsSNB genes*	Rice	System optimization	Wang F. et al. (2020)
	*BnALS1* and *BnALS3*	Oilseed rape	Herbicide resistance	Wu et al. (2020)
	Multiple sites at *OsALS1* gene	Rice	Facilitating the directed evolution of plant	Kuang et al. (2020)
	Multiple loci at *OsACC*	Rice	Herbicide resistance	Liu et al. (2020)
	Multiple targets trials	Rice	Prime genome editing optimization	Lin et al. (2020)
**Crop improvement**				
**Generating diversity and crop domestication**				
	Two sites at locule number (*Lc*) QTL + eight gRNAs targeting the promoters of *SlCLV3, S* and *SP*.	Tomato	Wild relative domestication	Rodríguez-Leal et al. (2017)
	Six targets at SP, SP5G, SlCLV3 and SlWUS	Tomato	Wild relative domestication	Li T. et al. (2018)
	Six targets at *SELF PRUNING, OVATE, FRUIT WEIGHT 2.2, LYCOPENE BETA-CYCLASE, MULTIFLORA* and *FASCIATED/YABBY* genes	Tomato	Domestication of wild *Solanum pimpinellifolium*.	Zsögön et al. (2018)
	*GS3, GW2*, and *GN1A*	Rice	Wild relative domestication (*Oryza glaberrima*)	Lacchini et al. (2020)
	Multiple	Rice	Wild relative domestication (*O. alta*)	Yu et al. (2021)
**Increasing crop yield potential**				
	*OsGW2, OsGW5*, and *OsTGW6*	Rice	Larger grain size	Xu et al. (2016)
	*OsGS3, OsGW2*, and *OsGn1a*	Rice	Larger grain size	Zhou et al. (2019)
	*GS3, GW2*, and *GN1A*	Rice	Increasing grain size	Lacchini et al. (2020)
**Improving crop product quality**				
	4 alleles of *GBSS*	Potato	Produce waxy potato	Andersson et al. (2017)
	*OVATE, FRUIT WEIGHT 2.2* and *LYCOPENE BETA CYCLASE*	Tomato	Fruit shape	Zsögön et al. (2018)
	Several sites at *St16DOX*	Potato	Reducing steroidal glycoalkaloids (SGAs)	Nakayasu et al. (2018)
	Two sites at *α-gliadin* gene	Wheat	Produce low-gluten wheat	Sánchez-León et al. (2018)
	*OsGS3, OsGW2*, and *OsGn1a*	Rice	Grain shape	Lacchini et al. (2020)
	*OsGS3, OsGW2*, and *OsGn1a*	Rice	Grain shape	Lacchini et al. (2020)
**Enhancing crop resistance/tolerance to biotic/abiotic stress**				
	*DIPM-1, DIPM-2*, and *DIPM-4*	Apple	Disease resistance	Malnoy et al. (2016)
	*OsSWEET11* and *OsSWEET14*	Rice	Generating broad spectrum disease resistance	Xu et al. (2019)
	*SWEET11, SWEET13* and *SWEET14*	Rice	Generating broad spectrum disease resistance	Oliva et al. (2019)
	*TMS5, Pi21*, and *Xa13*	Rice	Disease resistance and yield enhancement	Li S. et al. (2019)

### Knock-Out of Multiple Genes at Once

Loss of function or gene knockout has been widely investigated in plant genome-editing studies mainly because of the activation of the naturally occurring non-homologous end joining (NHEJ) repair pathway after Cas9 protein was reported to cause DSBs at the target DNA site. NHEJ is an error-prone repair mechanism that can generate insertion/deletion (Indels) of nucleotides near the cleavage site (Mao et al., 2013; Xie and Yang, 2013; Ma et al., 2015). These Indels mostly cause a frameshift mutation of the codon. Successful implementation of multiple gene knockouts mainly depends on the precise selection of the target loci.

In this regard, abundant studies have described multiple gene knockouts in different plant species for various purposes. Ali et al. (2015) explained the potential of CRISPR/Cas9 utilization in multiplexing two sgRNAs to knockout ssDNA viruses and expanded the possibilities of virus biological control in plants. In another attempt, Xu et al. generated a multiplex knockout to develop broad-spectrum disease resistance in rice by using a multiplex CRISPR/Cas9 system to alter the effector-binding elements (EBEs) of the bacterial blight TAL effector for two susceptibility genes, *OsSWEET11* and *OsSWEET14*, in rice cv. Kitaake (harboring the defeated *OsSWEET13*) (Xu et al., 2019). Furthermore, multiple gene knockouts have been involved in metabolite manipulations. In this regard, Kaur et al. designed a single gRNA to target the conserved regions of the two genes encoding RASPDS (Rasthali phytoene desaturase) of the banana cv. Rasthali (Kaur et al., 2018). PDS is one of the carotenoid biosynthesis pathway enzymes that limit the rate of carotenoid synthesis (Cazzonelli and Pogson, 2010; Bai et al., 2016), and its disruption impairs chlorophyll, carotenoid, and gibberellin biosynthesis, thereby producing albino plants. Moreover, multiplex knockout trials indicated the great promise in overcoming redundancy and polyploidy in double- and higher-order knockouts created by multiplex CRISPR/Cas9 in tomatoes (Li W. et al., 2016).

### Engineering-Efficient Plant Metabolic Pathways

Reducing or increasing the levels of specific metabolites through modulation of plant endogenous metabolic pathways has become achievable with the application of MGE. MGE has provided opportunities to investigate the roles of multiple genes in synthetic pathways in plants. For instance, using MGE, Ma et al. confirmed the role of *OsGSTU, OsMRP15, and OsAnP* genes in the anthocyanin synthesis pathway in rice (Ma et al., 2015). Moreover, the authors decreased the amylose content in rice by targeting three different sites in the *OsWaxy* gene.

Double and higher-order loss-of-function created by CRISPR/Cas9 offers great promise in modulating multiple genes in a single pathway or multiple pathways. Li et al. assessed the efficiency of multiplexing in tomatoes using the robust CRISPR/Cas9 system, as described by Ma et al. (2015) and Li X. et al. (2018) by targeting two loci in exons 6 and 7 of the tomato *PDS* gene (*slyPDS*). A strong photobleached phenotype was exhibited among the transgenic lines, and some T0 lines had concurrent homozygous modifications at both target sites. Subsequently, the authors used this system for metabolic engineering and manipulation of the gamma-aminobutyric acid (GABA) shunt metabolic pathway in tomatoes. They targeted five key genes in its pathway, *GABA-TP1, GABA-TP2, GABA-TP3, CAT9*, and *SSADH*. The researchers successfully edited five sites, whereas no editing events occurred in the third site, which was thought to be caused by the lack of CG content at this site. Quadruple mutant plants were obtained, which expressed the highest GABA levels in leaves, reaching almost 19-fold higher than that in wild-type plants. Li et al. discussed the relationship between various pathway components and the effects of mutations on certain components in the pathway. These results indicated the power of multiplex CRISPR/Cas9 as a tool for plant metabolic engineering and will improve our understanding of plant polygenic functional outcomes. In a bidirectional strategy, the same group targeted the loss-of-function of five different tomato genes using six gRNAs to increase the accumulation of lycopene while inhibiting its conversion to β- and α-carotene (Li X. et al., 2018).

Some plant metabolites, such as isoflavone, are controlled by multiple genes and involve specific pathways content (Li and Zhang, 2017). These compounds not only benefit human health but also play a critical role in the plant-environmental interactions for plant fitness in certain environments. Zhang et al. designed a single CRISPR/Cas9 binary vector to target three genes encoding key enzymes in the competing metabolic isoflavone biosynthesis pathway with the aim of increasing the isoflavone content in soybean plants (Zhang et al., 2020). The triple mutant (T3 homozygous plants) plant leaves had two times the level of isoflavone compared with that of wild-type plant leaves. The results of this study indicated the possibility of using MGE for loss-of-function competing pathway genes to increase the availability of specific plant substances or metabolites for another pathway.

### Transcriptional Regulation

Controlling gene expression has long been a goal of plant scientists for deciphering plant gene function and improving crop traits. CRISPR-mediated gene regulation, designed by engineering a nuclease-inactivated Cas9 (dCas9) protein with a transcriptional activation domain (VP64) or repressor domains (SRDX), has been very powerful in activating or repressing the endogenous expression of plant genes of interest (Lowder et al., 2017; Li Z. et al., 2019; Malzahn et al., 2019). With the help of gRNA, the dCas9 fusion protein can specifically bind to the promoter region and manipulate the expression of downstream genes.

To date, MGE-mediated transcription manipulation has mainly been focused on promoters implicated in gene repression, activation, and epigenetic modification. An increase of ~80-, 37-, and 192-fold was reported when targeting the promoter regions of the *WRKY30, RLP23*, and *CDG1* genes for simultaneous activation of their transcription in *Arabidopsis* (Li et al., 2017). Meanwhile, several gRNAs simultaneously targeting the same promoter in plants using dCas9-VP64 displayed synergistic effects (Lowder et al., 2015, 2018; Vazquez-Vilar et al., 2016). To determine the efficiency of CRISPR/Cas9 in controlling the gene expression of protein-coding and non-coding genes in *Arabidopsis*, additive transcription activation was observed by Lowder et al., who targeted three different sites in the promoter of the production of anthocyanin pigment1 gene (*AtPAP1*, encoding a transcription factor) and *miR319* (encoding a microRNA; Lowder et al., 2015). Gong et al. (2020) targeted two different genes, *OsWOX11* and *OsYUC1*, involved in crown root development and auxin biosynthesis in rice, respectively. They designed multiple gRNAs to target different sites in the promoter regions upstream of these genes, and the results indicated that targeting two different regions within 350 bp upstream of the TSS is most effective for dCas9-TV-based transcriptional activation.

With the flexibility and expandability of CRISPR/Cas9, it is possible to introduce several regulatory mutations of *cis*-regulatory elements (CREs), such as promoter enhancers and silencer sequences that regulate gene transcription, which could lead to a range of quantitative transcriptional and phenotypic changes. Rodríguez-Leal et al. (2017) provided a prime example of the utilization of a multiplex CRISPR/Cas9 system to fine-tune gene expression by targeting eight sites in the promoter regions of different tomato genes. The results have demonstrated that alteration of gene expression based on editing the CREs of key developmental transcription factors promises an attractive approach for generating desirable/beneficial regulatory variants, offering great potential for crop molecular breeding.

### Chromosomal Segment Restructuring

Chromosomal rearrangements include transposition, duplication, deletion, inversion, or translocation of nucleic acid segments (Gray, 2000). Chromosomal rearrangements occur regularly in plants (Udall et al., 2005; Szinay et al., 2012; Li J. et al., 2016; Zapata et al., 2016; Schmidt et al., 2019). The introduction of two DSBs in a single chromosome by multiple editing mainly leads to deletions (Siebert and Puchta, 2002) and sometimes inversions (Qi et al., 2013; Zhang et al., 2017), whereas two breaks on different plant chromosomes could lead to the occurrence of reciprocal translocation (Pacher et al., 2007).

Induction of deletions, inversions, and translocation using MGE has been employed in mammals and reviewed elsewhere (Cheong et al., 2018). Most of the reports about chromosomal segment restructuring in plants focused on introducing deletions. Deletions smaller than 100 bp were detected at high frequencies in the *N. benthamiana* T0 and *Arabidopsis* T2 populations, whereas deletions larger than 120 bp were still feasible, but at lower frequencies in *Arabidopsis* (Ordon et al., 2017). Durr et al. (2018) aimed to improve the propagation of deletions occurring in somatic cells by direct plant regeneration. Furthermore, different investigators have reported the generation of heritable deletions of genomic regions using CRISPR/Cas9 in *Arabidopsis, Medicago*, and rice (Zhou et al., 2014; Gao et al., 2015; Cermák et al., 2017; Wu et al., 2018). More recently, a unique study on inversion formation in *Arabidopsis* was reported, where an 18 kilobase (kb) segment was successfully inverted and transmitted to the next generation (Schmidt et al., 2019). The impact of these chromosome restructuring methods in the field of plant biology is remarkable because it is now feasible to redesign chromosomes according to specific needs (Schmidt et al., 2020).

### Multiplex Base Alterations

This application utilizes the CRISPR/Cas system to modify individual bases at multiple sites simultaneously for a gain of function. The establishment of this technology first relied on the fusion of cytosine deaminase or adenosine deaminase to the artificially mutated DNA nickase nCas9, which forms the cytosine editor (CBE) and adenine editor (ABE), respectively. In addition, the use of uracil glycosylase inhibitory protein (UGI) can increase the stability of uracil in DNA, resulting in enhanced editing efficiency. Combined with the multiple-targeting feature of sgRNAs, base editing can be achieved at multiple sites of the genome. Both CBE and ABE have been successfully applied in various model plants and crops (Chen et al., 2019). Most multiplexing base editing researches have focused on herbicide resistance (Shimatani et al., 2017; Kang et al., 2018; Li C. et al., 2018; Li Y. et al., 2019; Veillet et al., 2019; Wu et al., 2020). The reported maximum number of multiplexed sgRNAs in a single construct is four targets in rice, two targets at *OsWAXY*, and one at *OsCDC48* and *OsSNB* (Wang F. et al., 2020). Shimatani et al. (2017) reported a system with three sgRNAs targeting *ALS* and *FTIP1e* that was designed to generate double mutants resulting in the resistance to two herbicides. Moreover, using gRNA libraries, base editors can generate high-density substitutions in target regions, thus facilitating the directed evolution of plant genes (Kuang et al., 2020; Li et al., 2020; Liu et al., 2020). The development of different base-editing platforms was reviewed in detail in Mishra et al. (2020). Ren et al. developed a CRISPR/Cas9 toolkit, extensively enabling efficient base editing at desired sites in the rice genome (Ren et al., 2017). Multiple genomic sites could be targeted for single-base modification. These toolkits were further improved to increase the possibility of generating gain-of-function and loss-of-function mutants for several traits of agronomic importance through base editing (Ren et al., 2018). More recently, Li et al. (2020), created a system that enables the creation of saturated targeted endogenous mutagenesis editors (STEMEs) that allow simultaneous C to T and A to G conversions at specific target sites in plants, where both CBE and ABE are fused together with cas9 nickase and UGI.

A more advanced editing technique that efficiently produces all possible base conversions without the mandate of DSBs or donor DNA was developed, and it is called prime editing (Anzalone et al., 2019). This system uses a catalytically impaired Cas9 endonuclease fused to an engineered reverse transcriptase programmed with a prime editing guide RNA (pegRNA), allowing new genetic information to be written at a specific locus (Lin et al., 2020). Although the efficiency of prime editors is lower than that of base editors, the system is still a versatile and appealing new tool for creating all 12 types of single-point mutations, mixtures of several substitutions, and indels. Gao and her group used this system to develop mutant rice plants carrying G to T mutations, multinucleotide substitutions, and a number of desired nucleotide deletions (Lin et al., 2020). To date, it is very difficult to achieve these types of mutations using any other current editing tool.

## MGE in Crop Improvement

Multiplex genome-editing is an innovative approach that accelerates the crop improvement cycle and makes the expected outcome more feasible and achievable. This technology provides effective solutions to overcome the limitations of plant breeding approaches, particularly for providing required variants that cause characteristics desired by breeders at a fast pace without labor-intensive phenotypic and molecular evaluation of breeding germplasm. Although MGE applications are relatively new, the technology has been widely used to improve a diverse range of crops to gain in yield, quality, and nutritional value, herbicide resistance, and biotic and abiotic stress tolerance. In this section, we describe the revolution in crop improvement programs by introgression of this technology for editing plant genotypes to accomplish different breeding objectives.

### Generating Diversity and Crop Domestication Using MGE

Plant breeders aiming to develop new genotypes always seek to improve specific plant characteristics, and to achieve their goal, they unintentionally decrease genetic diversity among the crop genotypes. Furthermore, most of the studies on genetic diversity and QTLs depend on naturally occurring mutations that rarely occur in gene-regulatory regions such as promoters and upstream open-reading frames (uORFs) (Rodríguez-Leal et al., 2017), whereas traditional breeding often requires more effort to screen and utilize QTLs carrying favorable mutations.

The CRISPR/Cas9 may allow new genetic diversity to be generated by targeting specific genome loci, which may lead to the identification of new genes. In this context, Rodríguez-Leal et al. demonstrated that genome-editing tools can introduce new *cis*-regulatory alleles with the aim of identifying new alleles that provide beneficial quantitative traits (Rodríguez-Leal et al., 2017). A CRISPR/Cas9-based system was reported that targets the promoter sequence of genes to regulate three major productivity traits, namely, fruit size, inflorescence branching, and plant architecture in tomatoes. The results demonstrated a wide range of variations that could be achieved by altering the expression of the target genes.

Multiplex genome-editing further accelerates the domestication of crop wild relatives by modifying domestication genes. Domestication genes with marked effects on specific key phenotypes have been reported in barley (Komatsuda et al., 2007), maize (Wang H. et al., 2015), rice (Civán and Brown, 2017), and tomato (Li T. et al., 2018). Zsögön et al. designed a multiplex CRISPR–Cas9 strategy to target six genes in wild *Solanum pimpinellifolium* that are important for productivity in the present tomato crop breeding lines (Zsögön et al., 2018). The mutants were modified in their fruit number, size, shape, nutrient content, and plant architecture in a single generation and within a single transformation event. In the same context, with the aim of maintaining favorable characteristics of wild ancestors of *Solanum pimpinellifolium*, Li T. et al. (2018) edited four lines of four wild tomato accessions, all of which were resistant to bacterial spot disease and two of which were salt-tolerant, using the Csy4 multi-gRNA CRISPR/Cas9 system to target coding sequences, *cis*-regulatory regions, or uORFs of four genes associated with morphology, flower and fruit production, and ascorbic acid synthesis. The generated Cas9-free genotypes had a domesticated phenotype with parental disease resistance and salt tolerance. African rice, *Oryza glaberrima*, is a wild relative of the cultivated Asian rice *Oryza sativa*. African rice has specific characteristics to withstand diverse environmental stresses but has low yield ability. Lacchini et al. (2020) disrupted domestication loci of the cultivated African landrace Kabre, which resulted in mutants with significantly improved grain yield. Furthermore, domesticating allotetraploid rice was demonstrated by targeting six agronomically important traits of *O. alta* (an allotetraploid perennial wild rice) (Yu et al., 2021).

### Increasing Crop Yield Potential by MGE

High-yielding ability under different environments is the core trait behind developing new crop genotypes. Therefore, it is necessary to introduce multiple QTLs into an elite genotype to withstand different environmental stresses without yield penalties. These QTLs are generally located in different genetic backgrounds, and there is no certain guarantee that they will perform the best when they are transferred to other genetic backgrounds. Even in conventional crop breeding, this transfer requires multiple rounds of crossing and selection, which cost labor, time, and money, and linkage drag might often interrupt efforts with unfavored genes. MGE allows knocking out of multiple genes with negative effects on yield simultaneously from genotypes with specific resistance to environmental stress in a single generation. Xu et al. (2016) targeted three major genes, namely *OsGW2, OsGW5*, and *OsTGW6*, which negatively regulate rice grain weight. Double (gw5tgw6) and triple (gw2gw5tgw6) T1 mutants showed larger grain sizes than wild-type rice. Zhou et al. (2019) targeted negatively regulated genes for grain size, width, weight, and number, namely *OsGS3, OsGW2*, and *OsGn1a*, respectively, which directly affected grain yield, in three elite rice varieties. Similar results were obtained by Lacchini et al. (2020) after targeting the same genes (*OsGS3, OsGW2*, and *OsGn1a*) in a mutated African rice cv. Kabre with a disrupted *HTD1* gene (short stature compared to the wild type).

### Improving Crop Product Quality by MGE

Since yield is the most important trait for breeders, the adoption of new genotypes by farmers is strongly driven by crop quality traits (Dalton, 2004; Wang et al., 2012; Abdelrahman and Zhao, 2020). High-yielding varieties with improved quality are more acceptable to consumers, which in turn increases commercial value for the crop. Quality characteristics differ among crops; however, physical properties, eating quality, and nutritional properties are core traits.

Most of the studies that focus on the domestication of crop plants consider quality genes, among others, during planning for multiplex editing of different genes. Zsögön et al. (2018) aimed to create a novel genotype derived from tomato wild-type *S. pimpinellifolium* by generating loss-of-function mutations in six genes using MGE. Among the edited genes, three genes were related to the quality traits of fruit shape, fruit weight, and lycopene content (*OVATE, FRUIT WEIGHT 2.2*, and *LYCOPENE BETA CYCLASE*, respectively). Mutants with loss-of-function in the *OVATE* gene had an oval fruit shape, while no changes in fruit sizes were reported for *FRUIT WEIGHT 2.2* mutants. Meanwhile, mutants of *LYCOPENE BETA CYCLASE* had 100% more lycopene without a negative effect on β-carotene or lutein compared with the wild type. The level of lycopene, which is a kind of carotenoid, is one of the most important nutritional quality traits of tomato fruit. Li et al. successfully increased lycopene in tomato fruits to ~5.1-fold by using multiplex CRISPR/Cas9 genome editing of five genes that promote the synthesis of lycopene (*SGR1* gene) and genes that catalyze the cyclization of lycopene (*LCY-E, LCY-B1*, and *LCY-B2*, as well as *Blc*) (Li X. et al., 2018). The strategy was to promote lycopene biosynthesis while inhibiting its conversion into β-carotene and α-carotene. However, the increased number of mutant genes did not result in a concomitant increase in lycopene (Li X. et al., 2018). Instead, the *SGR1* single mutant exhibited the highest lycopene level followed by the plants with triple mutations in *SGR1, Blc*, and *LCY-E*.

Multiplex genome-editing has been successfully implemented for altering physical and nutritional quality properties. Lacchini et al. (2020) improved the grain shape of an *Oryza glaberrima* landrace when multiplexed editing of the *OsGS3, OsGW2*, and *OsGn1a* genes was performed. Kim et al. (2017) designed nine crRNAs to simultaneously target two homologous genes, *FATTY ACID DESATURASE 2-1A* (*FAD2-1A*, Glyma10g42470) and *FAD2-1B* (*Glyma20g24530*), in the soybean genome using CRISPR/Cas12a. *FAD2* encodes an enzyme that is responsible for the conversion of monounsaturated oleic fatty acid to a polyunsaturated linoleic fatty acid. Sánchez-León et al. (2018) designed two sgRNAs to target the α*-gliadin* gene using CRISPR/Cas9 to produce low-gluten wheat.

Moreover, several studies have reported improvements in the eating quality of crops using MGE. In potato (*Solanum tuberosum*), reducing steroidal glycoalkaloids (SGAs) is a requisite in breeding programs, as the accumulation of SGAs causes unfavorable taste. Nakayasu et al. (2018) multiplexed several sgRNAs to knock out a single gene (*St16DOX*; encoding a steroid 16α-hydroxylase in SGA biosynthesis) using hairy root culture and succeeded in developing SGA-free hairy roots of tetraploid potato (Nakayasu et al., 2018). In another study aiming to enhance the potato starch quality, Andersson et al. used three sgRNAs to target the four alleles of the potato granule-bound starch synthase (*GBSS*) gene to produce waxy potato (Andersson et al., 2017). Knocking out the four alleles caused a reduction in GBSS enzyme activity, leading to alteration in starch composition with a concomitant increase in the amylopectin/amylose ratio. Furthermore, mutants with only one functional allele still produced a significant amount of amylose.

### Enhancing Crop Resistance/Tolerance to Biotic/Abiotic Stress by MGE

Biotic and abiotic stress threatens food security, causing a reduction in crop production. Abiotic stress is more prominent during global environmental changes. Drought, salinity, and extreme temperatures are the main abiotic stressors that cause biological and biochemical effects on plant growth and development. Most of the gene editing for abiotic stress tolerance has been simplex targeting, i.e., one gene related to one constraint. Zafar et al. reviewed the efforts that have been made for the development of mutations for abiotic stress (Zafar et al., 2020). Ethylene-responsive factors (ERFs) are proteins that enhance plant tolerance to different abiotic stressors by activating stress-responsive genes. However, multiplex gene editing for biotic stress was more feasible because of the ease of targeting diseases susceptibility (*S*) genes, which confer a negative effect on disease resistance. Recently, Oliva et al. used CRISPR–Cas9-mediated genome editing to introduce mutations in the promoters of three *SWEET* genes (*SWEET11, SWEET13*, and *SWEET14*) of rice cv. Kitaake, IR64, and Ciherang-Sub1 for generating broad-spectrum bacterial blight resistance genotypes (Oliva et al., 2019). With the same objectives, Xu et al. (2019) generated mutations in two EBEs of the *S* genes *OsSWEET11* and *OsSWEET14* in rice cv. Kitaake. The resulting mutants were stable and consistently exhibited broad-spectrum resistance to most bacterial blight strains, with few exceptions in two generations of rice lines. Malnoy et al. (2016) mutated *DIPM-1, DIPM-2*, and *DIPM-4* to increase apple resistance to fire blight disease. Disease genes were also multiplexed with other traits to broaden the scope of the multiplexing objectives. Li S. et al. (2019) introduced mutations into the *TMS5, Pi21*, and *Xa13* genes in rice. Triple T1 mutants showed characteristics of thermosensitive genic male sterility (TGMS) with an enhanced blast and bacterial blight resistance. MGE will pave the way toward sustainable development of disease resistance by targeting more *S* genes in different plant species.

## Conclusion and Future Perspectives

Conventional plant breeding has limitations in meeting food security goals for ever-growing populations around the world. However, practical operations of screening germplasm for genetic variations, developing breeding populations, mutation breeding, and hybridization are usually labor-intensive and time-consuming. Genome editing is an innovative technology that is considered an important tool for plant biology studies and for crop improvements. Using MGE to simultaneously target different genomic regions has accelerated both basic and applied researches. Different applications of multiplex technology have been developed that allowed robust, routine CRISPR reagent delivery systems in plants. All these improvements made it easier to utilize MGE technology for enhancing plant biology knowledge by knocking out multiple genes to disrupt gene function and decipher their roles in metabolic pathways. Furthermore, multiplex CRISPR-mediated gene editing provides a gene regulation tool to activate or repress the expression of multiple genes simultaneously. With multiplex CRISPR/Cas9, it is possible to reformat and rearrange chromosomal segments based on the needs of biologists. The utilization of MGE may increase the effect of off-target occurrence as the number of targets increased (McCarty et al., 2020). Several online toolboxes for off-target detection and expectations have been developed and updated for eliminating the percentage of off-target occurrence (Cradick et al., 2014; Kang et al., 2020; Naeem et al., 2020; Störtz and Minary, 2021).

Applications of multiplex base editing have facilitated gain-of-function investigations; however, base editing generates mainly C–T and A–G conversions to date. More recently, prime editing has advanced base editing so that new genetic information can be written into specified DNA loci. This technology will expand the capabilities of MGE that may allow the gain-of-function of multiple genes simultaneously. With the identification and utilization of CRISPR systems, breeding schemes have become faster and more precise. Generating mutations with MGE allows diversity for selection and yield enhancement considering quality aspects. Improving specific traits without any other alteration to others may become possible in the near future.

Multiplex genome-editing will be enhanced by skipping long laborious and complicated plant regeneration and tissue culture steps by allowing CRISPR reagents to be delivered directly into plant meristems or pollen tissues, as reported by Maher et al. (2020), which presented a successful example of tissue culture-free plant gene editing. Furthermore, it is now also possible to write new genetic information to a specific locus in the genome (Lin et al., 2020). Despite diverging social perspectives, it is believed that multiplex CRISPR technology will play an important role in agricultural sustainability by developing resilient germplasm.

Implementation of MGE is necessary for understanding the dynamics of synthetic metabolic pathways. In addition, exploring enzyme functionalities in different pathways and their relationship with other omics information is crucial. Additionally, more attention should be devoted to utilizing this technology for the exploration of interactions among different metabolic pathways and gene regulatory, signal transduction, and protein-protein interaction networks in response to biotic and abiotic stress.

**Figure 2:**
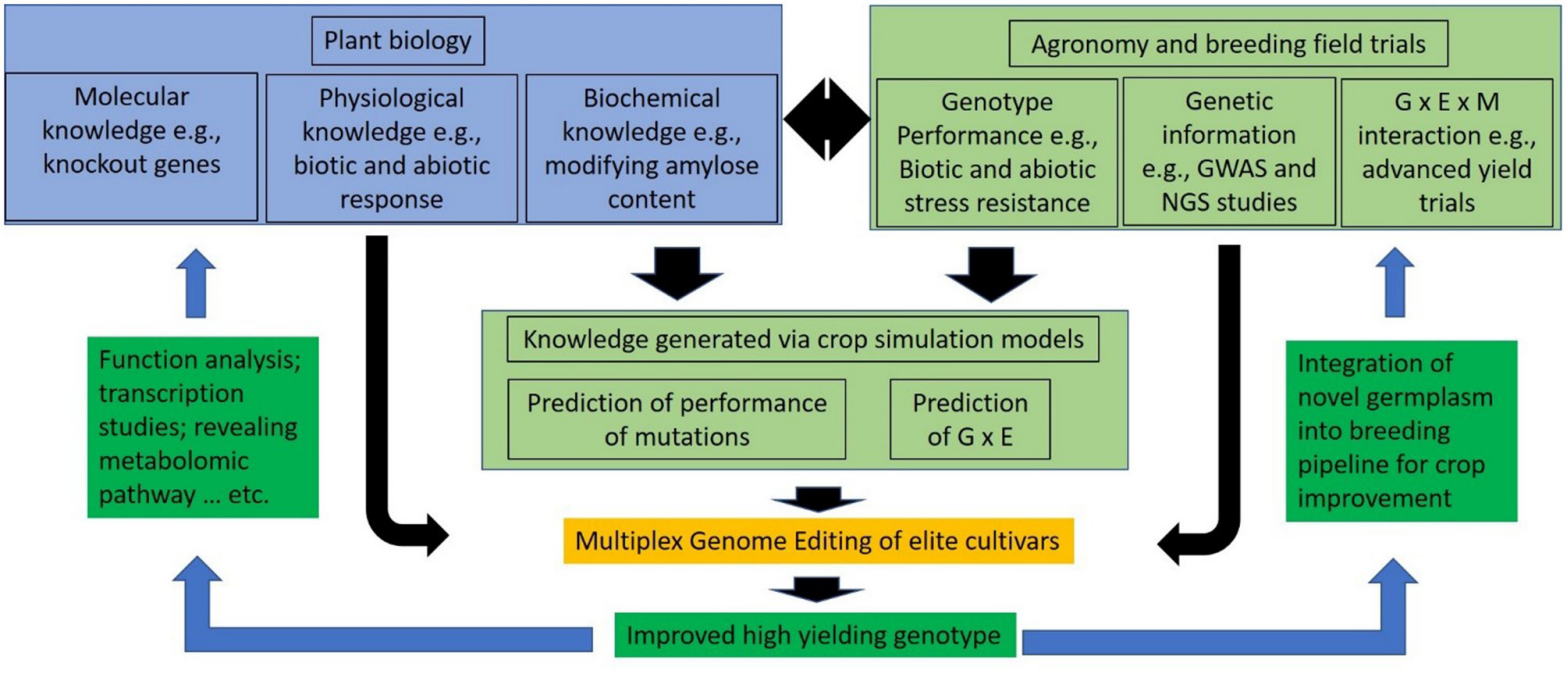
The role of multiplex genome editing (MGE) is explained: the development of novel germplasm is accelerated that either increases knowledge in plant biology or assists crop improvement programs.

Advanced knowledge regarding plant biology, agronomy, and breeding supports crop improvement programs. Through the utilization of such knowledge and new modeling approaches to predict the performance of the genetic variants (Wang H. et al., 2020), MGE will accelerate the development of elite crop cultivars with precise genomic modifications (Figure 2). MGE will increase and facilitate new plant biology discoveries and/or the development of new elite germplasm.

## Author Contributions

MA and KZ designed and discussed the content. MA and ZW wrote the first draft of the manuscript. JR and KZ critically revised the manuscript. All authors contributed to the article and approved the submitted version.

## Funding

This research was supported by grants from the Key R& D project in Sichuan Province of China (2021YFN0003 to KZ), the National Natural Science Foundation of China (U20A2035 to KZ), and the Talented Young Scientist Program of China (AiJi-18-062 to MA).

## Conflict of Interest

The authors declare that the research was conducted in the absence of any commercial or financial relationships that could be construed as a potential conflict of interest.

## Publisher's Note

All claims expressed in this article are solely those of the authors and do not necessarily represent those of their affiliated organizations, or those of the publisher, the editors and the reviewers. Any product that may be evaluated in this article, or claim that may be made by its manufacturer, is not guaranteed or endorsed by the publisher.
